# Application of Collagen Scaffold in Tissue Engineering: Recent Advances and New Perspectives

**DOI:** 10.3390/polym8020042

**Published:** 2016-02-04

**Authors:** Chanjuan Dong, Yonggang Lv

**Affiliations:** 1 Key Laboratory of Biorheological Science and Technology, Ministry of Education, Bioengineering College, Chongqing University, Chongqing 400044, China; happydcj@126.com; 2 Mechanobiology and Regenerative Medicine Laboratory, Bioengineering College, Chongqing University, Chongqing 400044, China

**Keywords:** collagen, biomaterial, scaffold, tissue engineering, extracellular matrix

## Abstract

Collagen is the main structural protein of most hard and soft tissues in animals and the human body, which plays an important role in maintaining the biological and structural integrity of the extracellular matrix (ECM) and provides physical support to tissues. Collagen can be extracted and purified from a variety of sources and offers low immunogenicity, a porous structure, good permeability, biocompatibility and biodegradability. Collagen scaffolds have been widely used in tissue engineering due to these excellent properties. However, the poor mechanical property of collagen scaffolds limits their applications to some extent. To overcome this shortcoming, collagen scaffolds can be cross-linked by chemical or physical methods or modified with natural/synthetic polymers or inorganic materials. Biochemical factors can also be introduced to the scaffold to further improve its biological activity. This review will summarize the structure and biological characteristics of collagen and introduce the preparation methods and modification strategies of collagen scaffolds. The typical application of a collagen scaffold in tissue engineering (including nerve, bone, cartilage, tendon, ligament, blood vessel and skin) will be further provided. The prospects and challenges about their future research and application will also be pointed out.

## 1. Introduction

Tissue engineering aims to reconstruct living tissues for replacement of damaged or lost tissue/organs, hoping to maintain, restore or enhance part or whole organ function of living organisms [1]. An ideal scaffold for tissue engineering is integral to achieve this goal. In natural tissue, extracellular matrix (ECM) is a collection of extracellular molecules secreted by cells that provides spatial and mechanical signals to cells and physical support to tissues [2,3]. It acts not only as a benign scaffold for arranging cells within the connective tissue, but also has a dynamic and flexible role that defines cellular behaviors and tissue function [2,4]. Therefore, it is a rational strategy to fabricate a scaffold that can mimic the ECM of damaged tissue or organ to repair it sequentially.

Collagen is the most abundant protein in the ECM and has been considered to be a group of proteins with a characteristic molecular structure—fibrillar structure, which contributes to the extracellular scaffolding [5]. That is to say, collagen plays an important role in maintaining the biological and structural integrity of ECM and provides physical support to tissues. Collagen possesses extensive sources (such as bone, cartilage, tendon, ligament, blood vessel, nerve, skin), as it is the main structural protein of most hard and soft tissues [6]. In addition, collagen offers low immunogenicity, a porous structure, permeability, good biocompatibility and biodegradability and has functions to regulate the morphology, adhesion, migration and differentiation of cells [7,8]. All of these good performances make this natural polymer seem to be a promising biomaterial for scaffolds in tissue engineering. However, the collagen scaffolds lack mechanical strength and structural stability upon hydration, which limit their applications in particular tissues. Intermolecular cross-linking of collagen scaffolds can be achieved by physical or chemical methods, which can improve the mechanical properties of the scaffold. Besides, blending collagen with other materials, such as natural, synthetic polymers and inorganic materials, is also frequently used to enhance the mechanical strength of collagen scaffolds. Meanwhile, biochemical factors could be added into or modified onto the scaffold selectively according to the damaged region to improve the cellular outcome.

In this review, we will summarize the characteristics of collagen and introduce the modification strategies of collagen scaffolds. Then, we focus on applications of the collagen scaffolds in nerve, bone/cartilage and tendon/ligament tissue engineering. The collagen scaffolds used for vascular grafts and skin substitutes are also presented. Finally, we discuss the directions and challenges in future research and the application of collagen-based scaffolds.

## 2. Characterization of Collagen as a Biomaterial

In the early in 1970s and 1980s, medical applications of biomaterials motivated researchers to focus their studies on collagen, and medical-grade collagen became easy to obtain [9]. In recent years, collagen has attracted more scientific interest with the development of booming tissue engineering technology [10,11,12,13], which relies mostly on fibrillar structure and excellent biological characteristics.

### 2.1. Structure of Collagen

Collagen represents the most abundant structural protein, accounting for approximately 30% of total body proteins in mammals [14]. To date, 28 different types of collagen have been identified [15], which could be defined into four major classes based on their compositional and structural characteristics [16]: (1) collagen with classically compact banded film structures, including types I, II and III collagens; (2) collagen with open fiber structures, like type IV and basement membrane collagen; (3) type V collagen and molecules containing the E and F chains; (4) collagen with a discontinuous triple helix. Collagen is a trimeric molecule consisting of three polypeptide α chains, which are numbered with Arabic numerals (Table 1). The α chains are woven together into a triple helix, which is the unique structural characteristic of the collagen family, to form homotrimers or heterotrimer. The triple helical sequences are comprised of Gly-X-Y repeats, X being frequently proline and Y often 4-hydroxyproline. Each collagen contains at least one triple helical domain (COL) located in the ECM, as well as non-collagenous (non-Gly-X-Y) regions (NC domains). Interspersing of the COL domains among NC domains makes the collagen multidomain protein. The NC domains participate in structural assembly and endow collagen with biological activities [14,17,18,19].

Collagen types I, II and III are the most common collagens and are called classical fibril-forming collagens. Collagen type I is the predominant collagen of most tissues in higher order animals. It consists of two α1 chains and one α2 chain, with a uniform size of 50 nm in diameter in native tissues [20]. Collagen type II is composed of three identical α1(II) chains and form fibrils less than 80 nm in diameter [21]. Collagen type III is found in limited quantities in association with collagen type I (about 10%) and is perceived as a minor contaminant of collagen type I prepared from skin [9]. It is composed of three α1(III) chains, forming fibrils with a variable size ranging from 30 to 130 nm in diameter [20,21].

### 2.2. Biological Characteristics of Collagen

Collagen is known for its poor immunogenic properties compared to other proteins [7]. Collagen antigenicity had been assumed to be non-existent on account of the similarity in the amino acid sequence among species before concerns on secondary effects caused by immune responses in the 1950s [9]. The major antigenic determinants in the collagen molecule are located in the non-helical telopeptide region. The other two are generated by the amino acid sequence in the helical region and triple helix structure of α chains. Removal of non-helical regions of the collagen molecule by protease treatment selectively can suppress its antigenicity to some extent. Cross-linking also can be introduced to reduce the antigenicity [7]. The cross-link formation can shield or modify major antigenic sites and, thus, reduce their capacity to interact with antibodies [22]. The immunogenic response could be avoided by choosing the right collagen source and proper experimental techniques.

**Table 1:** Collagen types, their chain composition and distribution (modified from [9,23,24]).

Type	Composition	Distribution
I	(α1(I))_2_α2(I)	Skin, tendon, ligament, bone, cornea, cartilage, large vessels, dermis, intestine, uterus, dentin, nerve
II	(α1(II))_3_	Cartilage, vitreous, nucleus pulposus, notochord
III	(α1(III))_3_	Large vessels, uterine wall, dermis, intestine, heart valve, gingival, skin, nerve
IV	(α1(IV))_2_α2(IV) α3(IV)α4(IV)α5(IV) (α5(IV))_2_α6(IV)	Basement membranes, nerve
V	α1(V)α2(V)α3(V) (α1(V))_2_α2(V) (α1(V))_3_	Cornea, placental membranes, bone, large vessels, hyaline cartilage, gingival, dermis, nerve
VI	α1(VI)α2(VI)α3(VI) α1(VI)α2(VI)α4(VI)	Descemet‘s membrane, skin, nucleus pulposus, heart muscle
VII	(α1(VII))_3_ (α1(VII))_2_α2(VII)	Skin, placenta, lung, cartilage, cornea, dermis, bladder
VIII	(α1(VIII))_3_ (α2(VIII))_3_ (α1(VIII))_2_α2(VIII)	Dermis, brain, heart, kidney
IX	α1(IX)α2(IX) α3(IX)	Cartilage, cornea, vitreous
X	(α1(X))_3_	Hypertrophic and mineralizing cartilage
XI	1α2α3α_1_ α1(XI)α2(XI)α3(XI)	Cartilage, intervertebral disc, vitreous humor
XII	(α1(XII))_3_	Tendon, ligament, dermis
XIII	Unknown	Skin, bone, intestinal mucosa, endothelial cells, dermis, eye, heart
XIV	(α1(XIV))_3_	Bone, dermis, cartilage
XV	Unknown	Capillaries, testis, kidney, heart,
XVI	Unknown	Dermis, kidney
XVII	(α1(XVII))_3_	Hemidesmosomes in epithelia
XVIII	Unknown	Basement membrane, liver
XIX	Unknown	Basement membrane
XX	Unknown	Cornea (chick)
XXI	Unknown	Stomach, kidney
XXII	Unknown	Tissue junctions
XXIII	Unknown	Heart, retina
XXIV	Unknown	Bone, cornea
XXV	Unknown	Brain, heart, testis
XXVI	Unknown	Testis, ovary
XXVII	Unknown	Cartilage
XXVIII	Unknown	Dermis, sciatic nerve, skin and calvaria. In zebrafish ^1^: nervous system, liver, thymus, muscle, intestine and skin

^1^ Collagen XXVIII in zebrafish was reported in 2015 [25].

Collagen has a relatively stable structure due to covalent cross-link formation among collagen fibrils. However, its protein nature determines the biodegradability. Collagen is broken down by catabolic processes in the tissues, involving enzymolysis of collagenase. Collagenase binds to triple helices at the surface and begins to degrade collagen fibrils from the outside. Then, molecules in the interior become accessible to the enzymes along with the degradation process, thus resulting in degradation of collagen fibrils from the outside to the inside. After triple helices are cracked, enzymes, such as non-specific proteinases and gelatinases, can facilitate further degradation of collagen molecules [9]. Besides that, pepsin, cathepsin and trypsin can accelerate collagen degradation *in vitro*. On the contrary, the degradation rate of collagen can be reduced by introducing cross-linking among fibrils according to the demand of collagen materials. In addition, collagen can induce platelet activation due to its participation in the intrinsic clotting cascade and is regarded as an attractive alternative to thrombin [26,27].

### 2.3. Extraction and Purification of Collagen

Collagen is abundant in sources because it is ubiquitous in many tissues or organs (Table 1). Tissues rich in fibrous collagen, such as dermis, tendon and bone, usually have a preference to be selected as sources to extract collagen. Studies have reported that purified collagen can be isolated from human peripheral nerve tissue [28] or human placenta [29], but the species are generally rat, bovine, porcine and sheep. Recently, fish collagen has attracted a great deal of attention, as it can be extracted and purified easily from wasted fish skins and bones [30]. Water-soluble collagen represents a small percentage of total collagen. The solubility property of collagen depends on the type of tissue and age of the donors. The most commonly-used solvents to extract collagen are neutral salt solution or dilute acetic acid. Strong alkali or enzymes are alternatives for insoluble collagen to cleave additional crosslinks [7,9].

### 2.4. Recombination of Collagen

The collagen extracted from animal tissues carries a risk of disease-causing contaminants and may cause allergic reactions, which limits its biomedical applications [31]. Recombinant human collagens provide a promising approach for mass production of collagen. Early in 1980, Uitto *et al.* [32] obtained type I and type III procollagen from human skin fibroblasts cultured under optimized conditions *in vitro*. The development of genetic engineering makes it possible to produce recombinant human collagens by host cells, such as yeast, bacteria, mammalian/insect cells, transgenic animals and transgenic plants [33]. Prolyl 4-hydroxyprolin (P4H) is a heterotetramer enzyme and is essential for the folding of the synthesized collagen polypeptide chains into triple helical molecules [34]. However, bacteria and yeast have no P4H activity, while insect cells have insufficient levels of activity. Therefore, the recombinant collagen polypeptide chains remain as non-triple helical and non-functional protein in these circumstances; the chains can only form unstable triple helices even at low temperature [34]. P4H can be introduced into the recombinant system to enable proline hydroxylation and the stability of the product [35].

## 3. Collagen-Based Scaffolds for Tissue Engineering

The material of scaffolds for tissue engineering can be any biomaterial that mimics one or multiple characteristics of the natural ECM [36], but is expected to function as a scaffold to replace natural collagen-based ECM. Much research has been reported on collagen, its denatured forms or collagen-based materials as biomaterials for scaffold fabrication in tissue engineering [2,10,21,37,38,39].

### 3.1. Pure Collagen Scaffold

Collagen is the main fibrous structural protein in the bodies of living organisms, and a collagen scaffold is beneficial to cells grown *in vivo* [39]. All of these merits determine the collagen scaffold to be a good platform for tissue repair and reconstruction. Bowlin *et al.* [20,40,41,42,43,44,45,46] has done extensive research on electrospun collagen scaffolds and has proven this technique to be an adequate way to support and mature cellular growth. Their research showed that collagen type I, II and III could form collagen fibers that are similar to or even fully reproduced the structural and biological properties of the natural collagen ECM under optimizing conditions. By electrospinning, collagen type I produced fibers exhibiting the 67-nm D-repeat banding pattern, which is a characteristic of native collagen [20]. Additionally, electrospun collagen exhibited the promotion of cell growth and penetration capacity. Lyophilization is another useful method to fabricate collagen scaffolds [47]. The collagen concentration in solutions determines the mechanical properties of the scaffold after lyophilization. Proper concentration could be chosen according to the implant position of the scaffold.

Despite the excellent biological properties of the pure collagen scaffold, it presents poor mechanical properties and structural stability. Physical treatment or chemical agents can be used to achieve intermolecular cross-linking of collagen, thus modifying the properties of the collagen scaffold. Ultraviolet (UV) irradiation, gamma radiation and dehydrothermal treatment (DHT) are the most commonly used physical treatments [48,49,50]. They could increase the mechanical properties of the collagen scaffold while reducing its solubility and absorption, but without any toxicity. The research of Takitoh *et al.* [49] even showed that gamma-cross-linked non-fibrillar collagen could promote elongation and osteogenic differentiation of mesenchymal stem cells (MSCs). Chemical modification is accomplished mainly by means of covalent of amine/imine linkage [51]. Glutaraldehyde (GA) is a synthetic cross-linking agent that has been widely used in the manufacturing of bioprosthesis. It produces collagen with a high degree of cross-linking, but with potential toxicity due to possible residue in the scaffold [52]. In addition, GA could induce an undesirable calcification of the scaffold after implantation. Another widely-used covalent cross-linking agent is 1-ethyl-3-(3-dimethylaminopropyl)-carbodiimide hydrochloride (EDC), used in the presence or absence of N-hydroxysuccinimide (NHS). Cross-linking is achieved by activation of carboxylic groups and subsequent formation of amide between amino and carbocylic groups of collagen. Importantly, as a zero-length cross-linking agent, EDC has not been reported to cause any cytotoxic reactions [42,53,54]. Genipin (GE) is a traditional Chinese herbal medicine derived from *Gardenia jasminoides*. It has been found to be a collagen cross-linking agent with high efficiency and negligible toxicity. However, its production of blue pigment limits its application in cornea tissue engineering [55,56].

### 3.2. Collagen/Natural Polymer Blend Scaffold

Cross-linking strategies of the pure collagen scaffold enhance the mechanical and structural properties, but may introduce negative effects on cellular response *in vivo*. Hence, a mixture of natural or synthetic polymers can be used to overcome the limitations of the monocomponent system. Natural polymers (such as chitosan, silk fibroin, hyaluronic acid, alginate, *etc.*) have been widely used in tissue engineering due to their similar features to native ECM. In this review, chitosan and silk fibroin were taken as representatives to elaborate applications of natural polymers in tissue engineering.

Chitosan has low toxicity, is non-immunogenic and biodegradable, which determines it as a great choice for biomedical applications. Additionally, chitosan is the only positively-charged biopolymer and is able to interact with structural molecules present in the ECM [57]. This unique cationic biopolymer can combine with other anionic biopolymer to form a two-component scaffold with optimum mechanical and biological properties. Therefore, a collagen-chitosan scaffold with a homogeneous structure can be fabricated through polyelectrolyte complexation of blended anionic collagen and cationic chitosan. The relationships between the component ratio or cross-linking methods and essential properties (such as morphology, stiffness, swelling, degradation and cytotoxicity) of the collagen-chitosan scaffold were systematically studied by Martínez *et al.* [58]. Yan *et al.* [59] showed that MSCs grow well on the chitosan-collagen porous scaffold with pseudopodia extending into the scaffold, indicating good cytocompatibility of MSCs with the scaffold. Then, the MSCs/scaffold composite was transplanted into the ischemic and infarct areas of rat. Double immunohistochemical staining showed differentiation of MSCs to neuron-like and astrocyte-like cells, suggesting a neuroprotective effect of the chitosan-collagen scaffold [59].

Silk fibroin is a natural macromolecular protein polymer with excellent biocompatibility, remarkable mechanical properties and biodegradability and has been concerned as a promising biomaterial for scaffold fabrication [60,61]. Research has shown that the contents and structure of silk fibroin nanofibers could modulate the morphology, adhesion, spread, migration and gene/protein expression level of olfactory ensheathing cells (OECs) [62]. Silk fibroin can be used to increase cell affinity to materials, to improve cell adhesion [63] and to enhance the mechanical properties of collagen-based materials [64]. This research demonstrated that a collagen-silk fibroin membrane loaded with 10 wt % of silk fibroin had the optimal mechanical properties and was beneficial to the proliferation of human corneal epithelial cells, reflecting the potential application of the collagen-silk fibroin composition in corneal tissue engineering [64]. Besides, other natural polymers, such as hyaluronic acid and alginate, are also commonly used to modify collagen-based scaffolds and reveal a promising prospect in tissue engineering applications [65,66,67].

### 3.3. Collagen/Synthetic Polymer Blend Scaffold

Blending collagen with natural polymers can improve the performance of collagen scaffolds, as described previously. Similarly, blending of collagen with synthetic polymers also makes it possible for scaffolds to perform both with optimal mechanical and biological properties in specific engineering applications. In this case, the synthetic polymer undertakes mechanical support to the structure of scaffolds, while collagen on the surface and inside of the scaffolds provides cell recognition signals, which is crucial for cell behaviors and development [19]. Scaffolds composed of collagen and synthetic polymers, such as poly (ε-caprolactone) (PCL), polylactic acid (PLA), poly (ethylene glycol) (PEG), polyglycolide (PGA), poly (lactide-*co*-glycolide) (PLGA) and polyvinyl alcohol (PVA), have been widely used for tissue engineering.

PCL is a non-toxic, low-cost, bioresorbable polymer with excellent mechanical properties and a slow degradation rate. Zhang *et al.* [68] developed PCL/collagen fibrous scaffolds and examined their characterizations and bioactivity. The fiber diameter of the scaffold ranged from 987 ± 274 nm to 689 ± 299 nm, decreasing with the increase of the collagen content. The PCL/collagen scaffold revealed low crystallinity, a small crystal size and a higher dehydration temperature (50 to 60 °C) than pure collagen (32.5 °C). Besides, the cellular behavior on the scaffold was investigated. Results indicated that the PCL/collagen scaffold could provide a suitable environment for adhesion and growth of L929 fibroblasts. Unidirectionally-oriented PCL/collagen nanofibers were fabricated using electrospinning, and the feasibility of the scaffold for implantable engineered muscle was examined [69]. The *in vitro* studies showed that the aligned composite nanofiber scaffold significantly induced human skeletal muscle cells’ alignment and myotube formation and may provide implantable functional muscle tissues to restore large skeletal muscle tissue defects.

Scaffolds composed of collagen and PLA have broad applications in tissue engineering. In a study done by Haaparanta *et al.* [70], a collagen/PLA hybrid scaffold with a highly three-dimensional (3D) porous structure was fabricated, in which PLA gives mechanical strength and collagen mimics the natural tissue environment of chondrocytes. The blended scaffold possessed open pores throughout the scaffold and showed a higher stiffness compared to the plain scaffold with only collagen. Moreover, the collagen/PLA scaffold showed the best penetration of chondrocytes into the scaffold among collagen/PLA, chitosan/PLA, collagen/chitosan/PLA blended scaffolds and plain scaffolds with only collagen or chitosan, indicating a promising application in cartilage tissue engineering. The fibrous network scaffold can be fabricated using poly-l-lactide (PLLA) and collagen type I by electrospinning [71]. In their study, hybrid PLLA/collagen (3:1 and 1:1) nanofibers were randomly oriented. The fiber diameter and the pore size of nanofibers decreased with the increase of collagen content, and the tensile modulus followed similar trends. When seeded with MSCs, cell proliferation on PLLA/collagen (1:1) scaffolds was found 256% higher than that on PLLA scaffolds after being cultured for 20 days. Furthermore, PLLA/collagen (1:1) scaffold promoted MSCs’ differentiation into endothelial cells (ECs) and expression of the EC-specific proteins. The orientation of fibers can affect cell migration profoundly. Lee *et al.* [72] found that MSCs cultured on the aligned PLLA fibers migrated 10.46-fold faster along the parallel orientation than along the perpendicular orientation of fibers. Therefore, it is feasible to fabricate aligned fibrous scaffolds to accelerate the process of tissue repair.

The development of tissue engineering relies on the evolution of scaffolds, which can provide a microenvironment mimicking native tissue and afford a proper biodegradation rate that matches with the neotissue formation rate. Under this remit, multicomponent scaffolds with multiple natural or synthetic polymers, such as collagen/chitosan/PLA [73], hyaluronic acid/collagen/PEG [74] and PCL/PLGA/collagen [75], exhibit their great application potential in vascular tissue engineering [73], repair of central or peripheral nervous systems [74] and regeneration of bone or liver tissue [75].

### 3.4. Collagen/Inorganic Hybrid Scaffold

Organic-inorganic composite materials have drawn much attention due to their ability to combine excellent properties of individual constituents. Hybridization can achieve tailor-made performances (such as morphology, stiffness, degradation) and meet various requirements in tissue engineering [76,77]. Several inorganic materials, such as hydroxyapatite (HA, Ca_10_(PO_4_)_6_(OH)_2_), silicate and β-tricalcium phosphate (β-TCP, Ca_3_(PO_4_)_2_), have been used in the construction of tissue engineering scaffolds.

HA is a bioactive ceramic material with high biocompatibility and is similar to natural bone tissue in chemical composition. HA has been widely used in bone tissue engineering due to its strong osteoinduction ability. Ngiam *et al.* [78] modified electrospun PLLA/collagen scaffolds with HA by an alternating soaking method. They found that HA improved the hydrophilicity of the scaffolds significantly and could enhance the cell capture efficiency of scaffolds to osteoblasts, which was beneficial to early cell capture of bone graft materials. The *in vitro* osteogenic potential of an electrospun PLLA/collagen/HA scaffold was also studied by Balaji Raghavendran *et al.* [79]. They indicated that the scaffold exhibited good cytocompatibility and superior osteoinductivity. Genes associated with osteogenic lineage of human MSCs on PLLA/collagen/HA scaffolds were upregulated significantly without the use of growth factors and specific medium, demonstrating that the hybrid scaffolds may be supportive for stem cell-based therapies for bone repair and reconstruction. β-TCP is well known as a bioabsorbable ceramic, its favorable biocompatibility and osteogenetic effect determine its applications for bone defect repairs [80,81]. Silicate is negatively charged, supporting its deposition on positively-charged collagen. Perumal *et al.* [82] discussed the influence of silica concentration on properties of collagen-silica composite scaffolds and found that the compressibility and biological stability of the scaffold linearly increased with the concentration of silica. The research indicated that only scaffolds with silica and collagen ratio less than one exhibited favorable surface biocompatibility. In conclusion, bioactive organic-inorganic composites inspired from the natural bone microstructure can be obtained by hybridization of collagen with HA, β-TCP or silica. Novel biomedical applications of collagen/inorganic hybrid scaffolds are available by combining other polymers or biological molecules with an appropriate proportion.

In recent years, carbon nanomaterials, such as carbon nanotube (CNT) and graphene, have gained considerable interest as potential solutions to some biomedical problems in tissue engineering [83]. CNTs can interact with collagen at the molecular level and relax the helical structure of collagen fibers. The addition of CNT increases the scaffold stiffness significantly due to its rigidity. Besides, CNT can enhance the functionality of collagen for biomedical applications. The CNT-enhanced collagen scaffolds can induce neural differentiation of stem cells effectively [84,85]. Graphene has favorable chemical, electrical and mechanical properties and has been demonstrated to be helpful for growth and differentiation of stem cells, including MSCs [86], neural stem cells (NSCs) [87] and induced pluripotent stem cells (iPSCs) [88]. The compressive strengths of collagen-based scaffolds blended with graphene or graphene oxide (GO) can be increased compared to the non-blended scaffold [89]. Shin *et al.* [90] fabricated a GO-PLGA-collagen hybrid fibrous scaffold with GO dispersed uniformly throughout the scaffold. The results indicated that the GO hybrid scaffold significantly enhanced attachment, proliferation and myogenic differentiation of C2C12 skeletal myoblasts, exhibiting superior bioactivity and biocompatibility.

### 3.5. Collagen Scaffold Modified with Growth Factors

Growth factors can regulate a variety of cellular processes; they are intercellular signaling molecules promoting cell migration, proliferation, differentiation and maturation depending on their type [91]. Our group [92] has reviewed nanofiber-based growth factor delivery for bone tissue engineering. The noted growth factors include bone morphogenetic protein (BMP), transforming growth factor beta (TGF-β), vascular endothelial growth factor (VEGF), fibroblast growth factor (FGF) and platelet-derived growth factor (PDGF). The loading methods of growth factors on nanofiber scaffolds can be classified into five categories: physical adsorption/encapsulation, co-axial electrospinning/emulsion electrospinning, encapsulated in micro/nanosphere, layer-by-layer (LbL) multilayer assembly and chemical immobilization by photo-immobilization, plasma treatment and click chemistry. All of these methods are applicable for growth factors modified on a collagen-based scaffold. For example, VEGF, FGF-2 and heparin-binding epidermal growth factor (HB-EGF) were loaded on collagen scaffold by physical adsorption through soaking scaffolds in phosphate buffered saline (PBS) containing growth factors [93]. Li *et al.* [94] fused stromal cell-derived factor-1α (SDF-1α) with a unique peptide of the collagen binding domain (CBD), so the CBD-SDF-1α can specifically bind to the collagen scaffold. In consideration of the importance of gradients of biological cues to nerve guidance and regeneration, SDF-1α gradated patterns on a collagen fibrous scaffold was fabricated. NSCs on the scaffold could sense the CBD-SDF1α gradient and tend to migrate toward regions with higher growth factor content [94].

Generally, there is an initial burst release of growth factors encapsulated in the scaffold, which is usually not effective and welcome. Hence, an appropriate loading method is crucial to the stable, sustainable and controllable release of growth factors on the scaffold.

## 4. Typical Applications of Collagen-Based Scaffold in Tissue Engineering

Collagen is the major component of ECM in many tissues or organs, which plays a key role in tissue development and in the maintenance of normal tissue architecture and function. In this section, several typical applications of collagen-based scaffolds in tissue engineering will be introduced, respectively.

### 4.1. Nerve Tissue

The nervous system plays a leading role in the human body, including modulating the function of each organ system and a variety of physiological processes in direct or indirect ways. Neurological diseases or nerve injuries can bring inconveniences to human life. Central and peripheral nervous system injuries can benefit from the use of tissue engineering strategies, *i.e.*, using the tissue engineering scaffold to facilitate the regeneration of injured nerves [95]. The collagen-based scaffold provides a good platform for nerve regeneration and repair.

The potential application of electrospun collagen nanofibers for spinal cord injury (SCI) was evaluated *in vitro* and *in vivo* by Liu *et al.* [96]. They took topographical signals, which provided contact guidance to cells or regrowing axons, into consideration and found that aligned fibers resulted in elongated astrocytes and the same orientation of neurite outgrowth from dorsal root ganglia (DRGs) with fiber axes. The *in vivo* study using a rat hemi-section model indicated the feasibility of fabricating 3D scaffolds using collagen fibers and their application potential for SCI repair.

It is an effective method to improve axonal regeneration by combining seeding cells with scaffolds. In research by Boecker *et al.* [97], a pre-differentiated MSC-seeded microstructured collagen nerve guide (Perimaix) was implanted in a 20-mm rat sciatic nerve defect. The seeded cells helped axons regenerate into the Perimaix nerve guide and were beneficial to myelination-related parameters. However, the results showed that pro-differentiation had no influence on functional recovery. The MSC-seeded Perimaix nerve guide could lead to an extent of functional recovery similar to autologous nerve transplantation, regardless of the differentiation status of MSCs. Similar conclusions were drawn by Bozkurt *et al.* by using Schwann cells as seeding cells [98]. As one of three elements of tissue engineering, growth factors are used to promote tissue recovery. Ciliary neurotrophic factor (CNTF), basic fibroblast growth factor (bFGF) and brain-derived neurotrophic factor (BDNF) are neuro-cytokines that can enhance nerve regeneration and accelerate functional recovery after nerve injury. Cui *et al.* [99] prepared functional collagen scaffolds by filling the collagen nerve conduit with linear ordered collagen scaffold (LOCS) combined with recombinant proteins CBD-CNTF, CBD-bFGF or CBD-CNTF + CBD-bFGF, respectively. The scaffolds were used to bridge a 35 mm-long facial nerve gap in minipig models to evaluate regeneration of peripheral facial nerves. Functional and histological observations at six months after surgery indicated that the functional scaffold accelerated nerve reconstruction, and the bi-factor group had a better effect than the single factor group. Previous research findings showed that external physical stimulation (such as electric [100], magnetic field [101], ultrasound [102] and laser [103]) could enhance the functional recovery of injured peripheral nerve. This physical stimulation could be introduced to combine with the scaffold to promote the repair rate of injured nerve.

### 4.2. Bone/Cartilage Tissue

Bone tissues are mainly composed of collagen type I and HA with a small amount of type V. Hence, collagens blended with nano-inorganic materials are more widely used to prepare scaffolds that mimic natural ECM of bone in bone repair.

**Figure 1:**
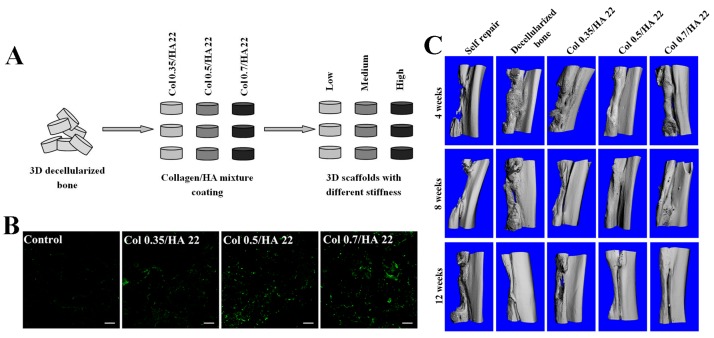
Fabrication of collagen/hydroxyapatite (HA) scaffolds and their application of bone repair. (**A**) Schematic diagram of the fabrication process of 3D scaffolds with different stiffness. Reprinted from [106]. Copyright 2015 with permission from American Chemical Society. (**B**) immunofluorescence staining of collagen type I on the scaffolds; scale bar: 200 μm. Reprinted from [106]. Copyright 2015 with permission from American Chemical Society. (**C**) μ-CT images of segmental radius at 4, 8 and 12 weeks [107]. Copyright 2015 with permission from Wiley.

Inzana *et al.* [11] fabricated collagen-calcium phosphate scaffolds using low temperature 3D printing. They maximized the cytocompatibility and mechanical strength of scaffolds by tailoring a certain concentration of phosphoric acid-based blind solution. Then, the scaffolds were implanted into a critically-sized murine femoral defect for nine weeks. Results indicated that the scaffolds were osteoconductive, and the scaffolds were partly broken down with new bone forming. In our laboratory, Chen *et al.* [104] fabricated an internally-structured collagen/HA scaffold by using collagen and HA, two major components of bone matrix. The study showed that collagen proportion could regulate the porosity and compressive modulus of the scaffold and further caused different cellular behaviors. The scaffold with a low collagen proportion (Col 0.35/HA 22) had good performance on MSCs’ viability and proliferation, while the scaffold with a high collagen proportion (Col 0.7/HA 22) had the best ability to motivate the osteogenic differentiation capability of MSCs. Considering that regulation of matrix mechanics on cellular behaviors and functions of stem cell in the 3D microenvironment [105], in another study [106], they fabricated scaffolds with different stiffness, but the same microstructure by coating decellularized bone with collagen/HA mixture in different collagen rations (Figure 1A,B). Their effects on adhesion and osteogenic differentiation of MSCs on the scaffolds were further studied. The *in vitro* results showed that the scaffolds could sustain the adhesion and growth of MSCs and promote their osteogenic differentiation. Subcutaneous implantation results indicated that the collagen/HA scaffolds could help to recruit MSCs from subcutaneous tissue and induced them to differentiate into osteoblasts. The study also pointed out that the scaffolds could provide a 3D environment for angiogenesis. Furthermore, the bone repair capacity of the scaffolds in the rabbit large bone defect model was evaluated. Results demonstrated that the cell-free collagen/HA scaffolds of proper stiffness combined with endogenous osteoprogenitor cells could increase the bone regeneration significantly (Figure 1C) [107].

Cartilage defects cause joint pain and loss of mobility [108]. Chondrocytes show low rates of regeneration due to their non-mobility and the absence of progenitor cells and vascular networks in the tissue [38]. MSCs have been commonly employed as a main source of seeding cells in cartilage tissue engineering. Many attempts have been made to induce cartilage differentiation of MSCs. Zheng *et al.* [108] used yarn-collagen/HA hybrid scaffolds to culture MSCs. Results showed that the scaffolds promoted orientation, adhesion and proliferation of MSCs. After being cultured for 21 days, MSCs on the hybrid scaffolds demonstrated upregulation of the collagen type II expression and increasing of glycosaminoglycan content. The results indicated that the yarn-collagen/HA scaffolds could enhance cartilage differentiation of MSCs. Muhonen *et al.* [109] prepared the collagen-PLA scaffold with recombinant human type II collagen. Then, they explored the potential of the scaffold in the repair of full-thickness cartilage lesions without additional cells compared to spontaneous healing and repair with a commercial porcine type I/III collagen membrane. Results showed that the scaffold group led to better repair of tissue and similar adverse subchondral bone reactions compared to the spontaneous healing group.

Autologous chondrocyte implantation (ACI) has made possible for hyaline articular cartilage regeneration, but the traditional ACI is plagued by complications caused by periosteal grafting [110]. Recently, much interest has been drawn to the combination of autologous chondrocytes with a tissue engineering scaffold to promote cartilage repair. The novel technology is called matrix-induced ACI (MACI), and the popular used matrix is artificial 3D collagen-matrix [111]. MACI comprises two surgical procedures: extraction, purification and expansion *in vitro* of chondrocytes; seeding of chondrocytes on a 3D matrix, which can subsequently be re-implanted. Griffin *et al.* [112] evaluated the performance of MACI using a collagen scaffold in an equine model after implantation for 53 weeks. The results demonstrated that cartilage defects receiving MCAI implants had 70% equilibrium modulus values of normal cartilage and had no statistical difference from normal tissue, representing a mechanical characterization of the MACI graft in a large animal model. MACI has been applied in clinical therapy for several years, and many clinical outcomes have been obtained. Basad *et al.* [113] treated patients with cartilage defects with MACI and evaluated their recovery conditions by Tegner (activity levels) and Lysholm (pain, stability, gait, clinical symptoms) scores at 6, 12, 24 and 60 months after surgery. The study showed that the Tegner score was improved from II to IV at 12 months and maintained to 60 months, while the Lysholm score was improved from 28.5 to 76.6 at 24 months and settled back to 75.5 at 60 months. The clinical outcomes indicated that MACI was a safe and effective technique for the majority of patients and highlighted MACI’s usefulness in surgical approaches for the treatment of damaged cartilage.

### 4.3. Tendon/Ligament Tissue

Tendons and ligaments are fibrous connective tissues, with collagen comprising 70% to 80% of their dry weight [15]. Both tendon and ligament have weak spontaneous regeneration ability and never totally recover from full-thickness lesions. The substantial donor site morbidity limits autograft applications for injured tissue and encourages the search for alternative solutions. Collagen scaffold provides an excellent way for tendon/ligament repair and regeneration.

An oriented multilamellar collagen I membrane was used to assess tendon regeneration properties and adverse reactions in an experimental animal model by Gigante *et al.* [114]. The collagen membrane was grafted into the central section of the patellar tendon (PT) of New Zealand white rabbit. The results showed good graft integration with native tendon and without any adverse side-effect. The study indicated that the collagen I membrane can serve as an effective tool for tendon defect repair. The electrospun fibers can mimic the fibrous structure of tendon and ligament, thus they can be used to fabricate scaffolds in tendon/ligament tissue repair. Cardwell *et al.* [115] studied the effects of the fiber diameter and orientation on tendon/ligament lineage differentiation of stem cells. Fibers consisting of small (< 1 μm), medium (1–2 μm) or large (> 2 μm) diameter with a random or aligned orientation were prepared by electrospinning technology. C3H10T1/2 stem cells were cultured on different fibrous scaffolds, and the cell morphology, growth and expression of tendon/ligament genes were then evaluated. The results declared that the fiber diameter has a greater influence on cellular behaviors than fiber alignment. Expression of tendon/ligament-related genes was suppressed on the fibrous scaffolds compared to spin-coated films. However, gene expression on the large-diameter fibers was higher than that on the medium-/small-diameter fibers. This result suggested a better prospect of the application of the larger diameter in tendon/ligament tissue engineering. In another study, thick collagen gel bundles with uniaxially-aligned fibrils and a sufficient size were fabricated [116]. The aligned structures improved mechanical and biological properties and resulted in elongation of cultured fibroblast, indicating potential applications in tendon/ligament reconstruction. The effects of the functional repair of this artificial tendon matrix still need further study. All of above research indicated that an oriented structure can promote the repairing efficiency of tendon/ligament due to the anisotropy of native tissues. Scaffolds that mimic features and functional properties of tissue *in vivo* actually provide an effective strategy for regeneration and repair of tendon/ligament.

### 4.4. Vascular Grafts

Numerous scaffolds possessing ideal characteristics for vascular grafts have been developed for clinical use. Lee *et al.* [117] developed a PCL/collagen fibrous scaffold by electrospinning. The PCL/collagen scaffold had the ability to resist high degrees of pressure and flow for a long time while providing a favorable environment for the growth of vascular cells. In their study, the PCL/collagen scaffold possessed sufficient elasticity (2.7 ± 1.2 MPa), appropriate tensile strength (4.0 ± 0.4 MPa) and higher burst pressure (4912 ± 155 mmHg) than that of pure PCL scaffolds (914 ± 130 mmHg) and native vessels. After being seeded with bovine ECs and smooth muscle cells (SMCs), confluent layers of ECs on the lumen and SMCs on the outer surface could be found in the scaffolds, indicating the excellent biocompatibility of the scaffolds. The finding suggested that PCL/collagen scaffolds in combination with vascular cells can be used to create tissue engineered blood vessels.

Vascular grafts may induce immediate thrombus after implantation due to their lack of healthy endothelium. Vascular endothelialization can reduce thrombosis, inhibit excessive hyperplasia of intima and significantly improve the long-term patency rate of artificial blood vessels. The development of endothelialization on cardiovascular materials surfaces was reviewed in detail by Liu *et al.* [118]. Zhu *et al.* [119] developed an intima layer scaffold for endothelialization by a freeze-drying process. The results found that the scaffolds with a 10:1 ratio content of collagen and hyaluronic acid possessed optimal performances, such as an interconnected porous network, better mechanical properties, low degradation and excellent biocompatibility. Study by Heo *et al.* [120] also indicated that collagen type IV immobilized onto electrospun nanofibers could modulate ECs’ function and enhance endothelialization of vascular grafts.

Considering the typical requirement of autologous cells, whose amplification *in vitro* is time consuming, cell-free vascular grafts have gained much attention. Koobatian *et al.* [121] engineered an acellular vessel using small intestinal submucosa and then modified it with heparin and VEGF. The tissue engineered vessel was implanted into the carotid artery model of sheep. Host cells infiltrated into the vascular significantly as early as one week after implantation, and a confluent functional endothelium was found at one month. After three months, the endothelium aligned in the flow direction, and circumferentially-aligned SMCs consisted of the medial layer, indicating successful endothelialization and remodeling of the cell-free vascular graft. Combining the cell-free vascular graft with endogenous ECs represents a great progress in vascular tissue engineering. Future research directions may be focused on the development of vascular material and immobilization of growth factor.

### 4.5. Skin

Skin is the body’s largest organ, composed of epidermis, dermis and hypodermis layers. The skin wound is an old and pendent problem in the surgical field. Skin substitutes can suppress the formation of the fibrotic scar. However, the commonly-used skin substitutes, such as allografts and autografts, cannot solve the problems caused by extensive or full-thickness skin loss, which would lead to skin dysfunction. Therefore, many researchers have directed their attention to tissue engineering, hoping to promote regeneration of skin.

Collagen-based scaffolds have a distinct advantage in skin tissue engineering, as collagen type I makes up 70% to 80% of the dry weight of dermis [2]. Ma *et al.* [122] found that GA-treated collagen/chitosan scaffolds had good cytocompatibility and could promote cell infiltration and proliferation effectively. Besides, the scaffolds could accelerate infiltration of the fibroblasts from the surrounding tissue *in vivo*, indicating their potential application for dermal repair. Rho *et al.* [123] developed fibrous collagen scaffolds and studied their effects on human keratinocytes and application in skin tissue engineering. Results showed that the scaffolds promoted cell adhesion and spreading of human keratinocytes *in vitro*. In open wound healing tests, the collagen scaffold accelerated the disappearance of the surface tissue debris and proliferation of fibroblasts and young capillaries in the early stage of wound healing. The fish-sourced collagen has similar characteristics as conventional collagens and carries no disease risk. The application of fish collagen-based scaffolds in skin tissue engineering has been studied recently [124,125]. Many organic/inorganic materials can be used to strengthen the properties of collagen scaffolds in scientific research, but the materials approved by the U.S. Food and Drug Administration (FDA) for clinical applications are limited. The FDA has approved only collagen, hyaluronic acid, PLLA, HA and non-biodegradable polymethylmethacrylate (PMMA) beads used as dermal fillers since July 2015 [126]. Much research should be done to meet the clinical requirements of therapeutic applications.

## 5. Conclusions

Collagen-based scaffolds have been proven to possess excellent biocompatibility and sufficient mechanical properties and have gained great achievements in tissue engineering. As shown in this review, collagen-based scaffolds are widely used in tissue engineering, including nerve, bone, cartilage, tendon/ligament, vascular grafts and skin, in which all provide obvious promotion functions to tissue repair, both *in vitro* and *in vivo*. However, the state of the art for collagen-based scaffold clinical application is limited. There is a long way to go from bench to bedside. More efforts should be devoted to the construction of scaffolds with optimum structures and properties.

A collagen scaffold working well in one specific tissue may have a poor effect on another one, as tissue or organ *in vivo* has its own unique microenvironment. As stem cells on substrates with various stiffness can differentiate into the corresponding cell lineage, mechanical and biological properties should better mimic those of the native tissue. Similarly, surface properties (chemical composition, topography), mechanical properties, electrical properties and morphological properties of the scaffold are all critical cues for directing cellular behavior and fate; they reveal an issue about the parameters that researchers must take into account when they intend to improve scaffold performance. Besides, the microenvironment is dynamically changed through the phases of tissue repair. An understanding of the changing process would be helpful for the construction of scaffolds.

It also should be pointed out that only some typical applications of collagen-based scaffolds were presented in the present review. Recently, collagen scaffolds used for corneal wound healing [127] or tympanic member repair [128,129] achieved good repair effects, which indicate the possibility to open up some new areas for collagen scaffold use. On the other side, the widespread application of collagen scaffolds brings a problem about excessive consumption of collagen. Collagen extracted from animals is limited in amount; thus, recombinant collagen plays an important role in the mass production of collagen. Much work should be done for the improvement of the recombinant system and exploration of new types of collagen, as some recombinant collagen polypeptide chains cannot form a stable triple helical structure without sufficient enzyme activity. In future research, researchers should pay more attention not only to fabrication of collagen-based scaffolds with better performance, but also to exploration of collagen that mimics natural collagen, both in structure and biological properties.
